# Bilateral uterine vessel ligation as a model of intrauterine growth restriction in mice

**DOI:** 10.1186/1477-7827-12-62

**Published:** 2014-07-08

**Authors:** Mathilde Janot, Marie-Laure Cortes-Dubly, Stéphane Rodriguez, Uyen Huynh-Do

**Affiliations:** 1 Department of Nephrology, Hypertension and Clinical Pharmacology, Inselspital, University of Bern Medical School, Bern, Switzerland; 2 Department of Clinical Research, Inselspital, University of Bern Medical School, Bern, Switzerland; 3 Charles River France, Les Oncins, BP 0109, 69592 L’Arbresle cedex, France

**Keywords:** Uterine growth restriction, Uterine vessel ligation, Pregnancy, Fetus, Placenta, Mouse model

## Abstract

**Background:**

Intrauterine Growth Restriction (IUGR) occurs in up to 10% of pregnancies and is considered as a major risk to develop various diseases in adulthood, such as cardiovascular diseases, insulin resistance, hypertension or end stage kidney disease. Several IUGR models have been developed in order to understand the biological processes linked to fetal growth retardation, most of them being rat or mouse models and nutritional models. In order to reproduce altered placental flow, surgical models have also been developed, and among them bilateral uterine ligation has been frequently used. Nevertheless, this model has never been developed in the mouse, although murine tools display multiple advantages for biological research. The aim of this work was therefore to develop a mouse model of bilateral uterine ligation as a surgical model of IUGR.

**Results:**

In this report, we describe the set up and experimental data obtained from three different protocols (P1, P2, P3) of bilateral uterine vessel ligation in the mouse. Ligation was either performed at the cervical end of each uterine horn (P1) or at the central part of each uterine horn (P2 and P3). Time of surgery was E16 (P1), E17 (P2) or E16.5 (P3). Mortality, maternal weight and abortion parameters were recorded, as well as placentas weights, fetal resorption, viability, fetal weight and size. Results showed that P1 in test animals led to IUGR but was also accompanied with high mortality rate of mothers (50%), low viability of fetuses (8%) and high resorption rate (25%). P2 and P3 improved most of these parameters (decreased mortality and improved pregnancy outcomes; improved fetal viability to 90% and 27%, respectively) nevertheless P2 was not associated to IUGR contrary to P3. Thus P3 experimental conditions enable IUGR with better pregnancy and fetuses outcomes parameters that allow its use in experimental studies.

**Conclusions:**

Our results show that bilateral uterine artery ligation according to the protocol we have developed and validated can be used as a surgical mouse model of IUGR.

## Background

Intrauterine growth restriction (IUGR) refers to a condition in which a fetus is unable to reach its genetically determined potential size. It can result in a baby being Small for Gestational Age (SGA), the fetal weight being below the 10th percentile for the gestational age. SGA group also includes fetuses that are constitutionally but not pathologically small. IUGR can either lead to symmetric growth restriction, where fetal entire body is proportionally small, or asymmetric growth restriction, where growth of vital organs such as the brain and heart is maintained at the expense of the liver, muscle and fat consequently to insufficient nutrient delivery. This state can evolve to symmetric growth restriction, if the factor responsible for growth restriction is severe or sustained long enough [1]. IUGR is thus a major cause of perinatal morbidity and mortality [2], and is of high clinical relevance since it represents up to 10% of all pregnancies [3]. This phenomenon corresponds to a fetal adaptation to an inadequate supply of oxygen and/or nutrients. Various studies have shown that an adverse uterine environment may be a cause of diseases in adulthood, such as hypertension, cardiovascular diseases, insulin resistance or end stage kidney disease [4-7]; thus IUGR constitutes an important risk factor for these diseases. Consequently, several animal models have been developed to study the mechanisms of IUGR. Most of these studies have been performed in rodents (principally in rats); others implicated bigger animals such as sheep, pigs, monkeys or lambs [8]. A widely used experimental model consists in altering nutritional parameters during pregnancy with caloric restriction, low protein diet or iron deficiency [8]. This model is particularly interesting to reproduce malnutrition conditions that are a major cause of fetal growth retardation in the world. Other common models consist in exposing pregnant animals to hypoxic conditions [9] or surgical interventions, which themselves lead to hypoxia by decreasing placental flow and thus affect both placenta and fetus. These surgical models are relevant to study human pregnancy in developed countries, where altered placental flow is the main mechanism implicated in IUGR [8].

The most frequently used surgical model corresponds to bilateral uterine artery ligation in sheep, rabbits or rats [8,10,11]. Ligation of uterine arteries is performed, leading to ischemia. But despite its frequent experimental use, this model shows several weaknesses. First, this surgery is accompanied by high maternal mortality, fetal resorption and variation in fetal weights [12,13]. More importantly, a recent carefully performed study showed experimentally that bilateral ligation of the uterine arteries in rats didn’t reproducibly lead to IUGR, while a meta-analysis of the literature indicated a bias in publications where pups with IUGR were selected [10]. As a consequence of the problems mentioned above, (high rate of fetal resorption in smaller animal species) there has been almost no studies using this surgical technique in mouse, which is however the most used animal model by the scientific community. Notably, many genetic mouse models have been described and constitute fundamental tools to understand several biological and pathological mechanisms.

The aim of this study was therefore to set up and validate a model of fetal growth retardation in pregnant C57BL6/J mice induced by bilateral uterine vessel ligation. Pregnancy outcomes parameters such as mortality, weight and abortion were checked; fetal weight and size were measured, as well as fetal resorption, viability and placentas weights. Our results showed that bilateral uterine vessel ligation is indeed an effective method to induce IUGR in the mouse model.

## Methods

### Animals

Pregnant C57BL6/J mice from Charles River France laboratories were group-housed prior to surgery, then housed in individual cages after surgery. Mating was done in Charles River France laboratories and the mating date (half day) was recorded precisely. Mice were maintained in 12 h light/dark cycles and allowed free access to food and water and were then transferred to the site of surgery in Charles River's laboratories. All animal experiments were performed according to European and Swiss Animal Welfare laws and were reviewed and approved by the animal ethics committee from Charles River Laboratories, France.

### Surgery and study design

**Figure 1:**
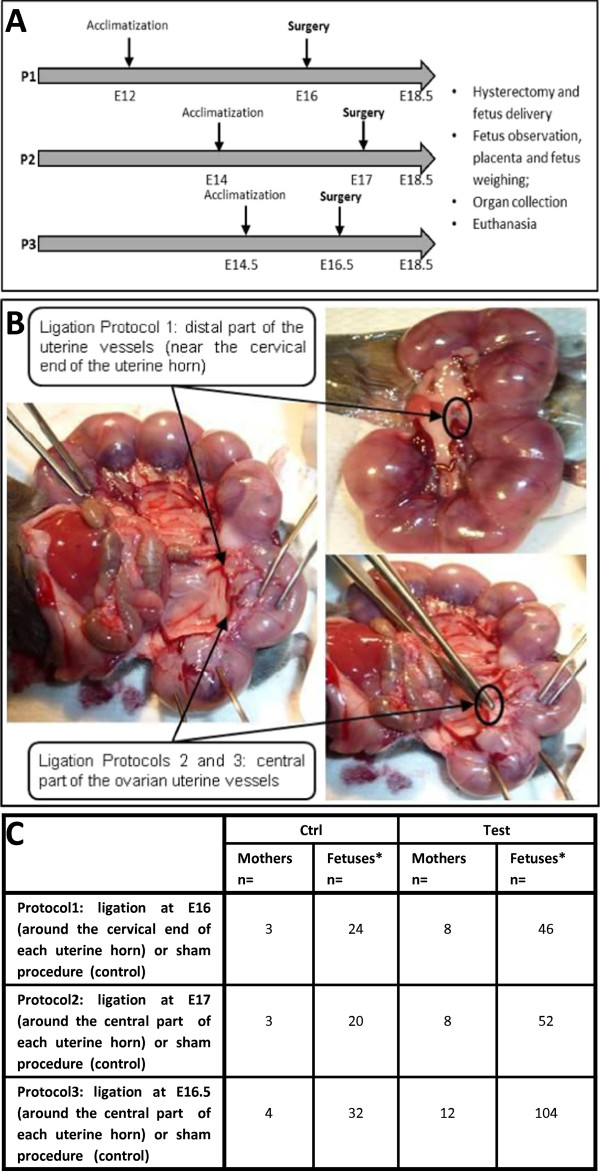
**Description of the three protocols of bilateral uterine ligation experiments. A**. Study scheme of the experiments. After acclimatization, surgery was performed on pregnant mice at E16 (P1), E17 (P2) or E16.5 (P3). Fetuses were extracted before delivery, at E18.5, preceding general observation, weighing, euthanasia and organ collection. **B**. Ligation localization. In P1, ligation was performed in test animals around the distal part of the uterine vessels, near the cervical end of each uterine horn; in P2 and P3 ligation was performed in test animals around the central part of each uterine horn, generally between fetus 2 and 3. Control animals underwent sham procedure. **C**. Number of pregnant mice and fetuses (at surgery) used in the three different protocols. *P, Protocol.*

In order to set up the most suitable mouse model of IUGR, chronologically three different protocols were followed (P1, P2, P3 - Figure 1A). The design of P2 and P3 was modified according to the results of P1 and P2 respectively, especially in terms of surgical technique (ligation site and type of suture at ligation), analgesia and time between surgery and termination. In P1, surgery was performed on embryonic day 16 (E16) while surgery from P2 and 3 were performed at E17 and E16.5, respectively. E16.5 surgery was performed A.M while E17 surgery was performed P.M. In P1, animals received a pre-operative subcutaneous injection of an analgesic buprenorphine (Buprecare®, 0.05 mg/kg) which was associated by abdominal suture pulling out for several mice. This phenomena was considered to be associated with pruritus induced by buprenorphine use (secondary effect already observed after subcutaneous injection of buprenorphine in rats). In P2 and P3, Flunixine meglumine (Finadyne®, 2.5 mg/kg), an analgesic (NSAID) without any impact on pregnancy, was thus chosen to replace buprenorphine. Animals then underwent general anesthesia with isoflurane. A midline laparotomy was performed in order to expose the uterine horns and their vascularization, fetuses were counted and surgery was performed (Figure 1A- C). At P1, ligation site was chosen as described in the literature [11]. The ligation (Vicryl 5–0 Ethicon®) of the uterine vessels (containing the uterine artery and vein) was performed distally near the cervical end of each uterine horn. This ligation induced a significant reduction of the blood flow as the blood flow from the iliac artery was completely stopped. The consequence of this blood flow reduction was a very low viability of the fetuses and a high rate of partial abortion (defined as the pre-term loss and expulsion of a fetus or embryo) in the test group. In P2, ligation site was modified in order to obtain a moderate blood flow reduction as compared with P1 and then a higher viability of the fetuses and lower rate of partial abortion in the test group. The uterine vessels were ligated in P2 and P3 at the central part of each uterine horn (generally between fetus n°2 and 3 (P2 and P3, Figure 1B) so that the blood flow from both the iliac and ovarian arteries was still existing. The results of P2 confirmed this hypothesis: a higher rate of fetus’ viability and a lower rate of partial abortion were obtained in the test group. The type of suture (Vicryl in P1 and Ethibond in P2 and P3) was modified in P2 and P3 to Ethibond 5–0, Ethicon® due to surgical preferences. Control group animals underwent sham procedure without ligation. After the uterus was placed back into the abdominal cavity, animals received Lidocaïne (Xylovet®, 3.5 mg/kg) at the incision site (P1, administration of Lidocaïne before closure of the abdominal wall; P2 and P3, the abdominal wall was closed and animals received a subcutaneous injection of Lidocaïne). Until complete recovery, oxygen was delivered to females that were maintained on heating pads and kept under close observation. An analgesic subcutaneous injection of flunixine meglumine (Finadyne®, 2.5 mg/kg) (P2 and P3) or buprenorphine (Buprecare®, 0.05 mg/kg) (P1) was administered at the end of the day of surgery and the next day. Females underwent caesarian section at E18.5 and were then sacrificed.

### Calculation of fetal resorption and viability rates

Fetal resorption rate was calculated as the number of fetuses counted at the time of surgery minus the number of fetuses found at E18.5 at the time of caesarian section. Results are expressed as a percentage of fetuses counted at the time of surgery. Fetal viability was calculated from the total number of fetuses found at termination (E18.5 – in the uterus or in the bedding). Viability was evaluated using macroscopic parameters such as coloration (normal pink/purple *vs*. brown, green, black), body consistency (normal, dry or soft), size, or body malformation. Data are presented as a percentage of viable fetuses counted at termination.

### Fetal weight, size measurements and tissue collection

On E18.5, animals underwent isoflurane anesthesia and hysterectomy. Number and localization of fetuses in each uterine horn was performed, dams were sacrificed by pentobarbital intracardiac injection. Placentas were collected and weighed. Size of the fetuses was calculated by multiplying the cranio-caudal and dorso-ventral distances of the fetuses. After macroscopic observation (viability, color, size) and weighing, fetuses underwent euthanasia by decapitation. Kidneys, liver, spleen, hearts and lungs were collected for further macroscopic observations including shape, coloration, consistency, differentiation and presence of blood spots. Percentage of fetuses with IUGR was calculated using the total number of fetuses from control or test animals and the number of fetuses meeting IUGR conditions (2 SD below the mean weight of control fetuses).

### Glomerular counting

Kidney tissue for estimation of glomeruli number was fixed with 2% paraformaldehyde, embedded in paraffin and sectioned at 8 μm. Total number of glomeruli in a total volume of 49,6 μm^3^ was estimated on FFPE kidney sections stained with H&E following the physical fractionator stereologic technique [14].

### Statistics

Data are presented as mean ± SD. Unpaired *t* test based on average per litter was used to analyze differences observed between two groups. Data were considered significant when *P* value < 0.05. Significance levels were ***, *P* < 0.001; **, *P* < 0.005; *, *P* < 0.05. All statistics were analyzed with the GraphPad Prism5 package.

## Results

### Follow-up of operated mice and pregnancy outcomes

**Figure 2:**
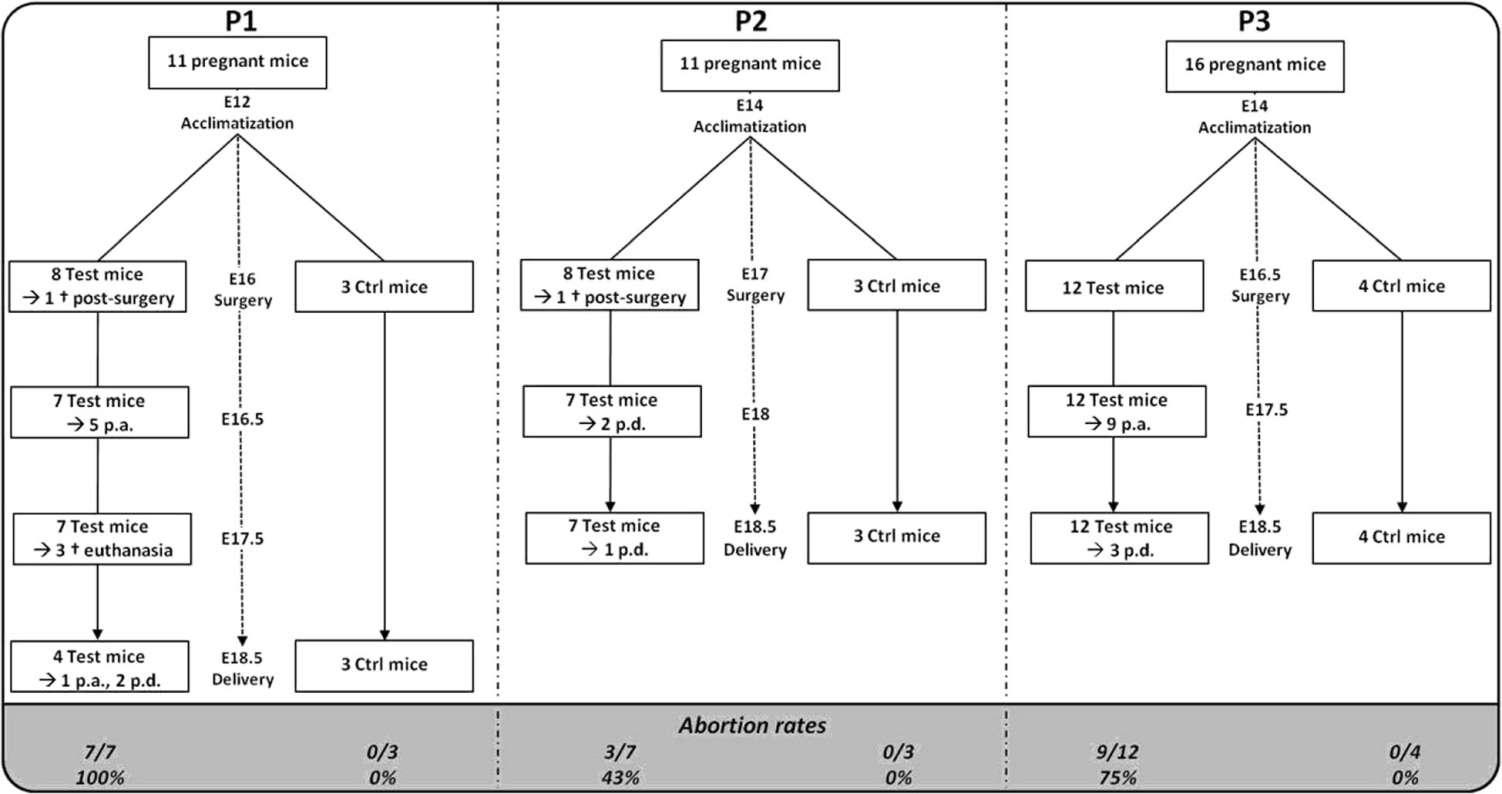
**Study outlines from P1, P2 and P3.** Study outlines from P1, P2 and P3 with number of animals used in each protocol and pregnancy outcomes (p.a., partial abortion; p.d., partial delivery). Number and percentage of mice that presented some aborted fetuses (partial abortion) are highlighted under each diagram; P1 was associated to the worst pregnancy outcomes.

The follow-up of operated animals until termination day (E18.5) indicated major differences in pregnancy outcomes depending on the considered protocol. Indeed, P1 was associated with the most severe impact as all mice from the test group presented some aborted fetuses (partial abortion) (Figure 2). P2 presented better maternal parameters with 43% of mice undergoing partial abortion, while P3 showed an intermediate situation (75% of partial abortion). Weight loss was also more severe in P1 and P3 than in P2 (Table 1), which nevertheless led to weight loss in both test and control groups: in P2, test mice presented a decrease of 18% of their weight over 1.5 day after surgery; control mice also lost weight with a decrease of 6% but with stabilization at the end of experiment (Table 1). In P1, test animals lost around 21% of their body mass, in contrast to what was observed in control animals which gained around 9% of their body mass over 2.5 days. Weight loss in test animals from P3 was similar to what was observed in P1 with a decrease of 22%; control mice weight was stable with an overall decrease of 1% (SD 3%) over 2 days but with an increased weight after 1 day of surgery. On the termination day (E18.5), macroscopic observations also showed the impact of surgery on the uterus with abnormal colorations of the surfaces of uterine horns in 57% (P1, P2) to 58% (P3) of test animals (data not shown).

**Table 1:** Comparison of general parameters from P1, P2 and P3

**Parameters**		**P1**		**P2**		**P3**	
		**Ctrl**	**Test**	**Ctrl**	**Test**	**Ctrl**	**Test**
***Pregnancy outcomes***	**Weight monitoring**	+ 9	−21	−6	−18	−1	−22
		± 1%	± 5%	± 1%	±8%	+/−3%	+/−10%
	**Abortion**	0%	100%	0%	43%	0%	75%
	**Mean number of fetuses**						
	at surgery (D0*)	8 +/− 1	7 +/− 1	7 +/− 1	7 +/− 1	8 +/− 0	9 + −/ 1
	**at extraction (E18.5)**	8 +/− 1	4 +/− 2	7 +/− 1	6 +/− 2	8 +/− 0	4 +/− 2
***Fetal data***	**Fetal resorption**	0	25	0	0	0	37
***(E18.5)***		+/− 0%	+/−30%	+/−0%	+/−0%	+/−0%	+/−33%
	**Fetal viabiliy**	92	8	90	59.	100	27
		+/−6%	+/−14%	+/−7%	+/−22%	+/−0%	+/−33%
	**Mean weight of fetuses**	0.92	0.45	0.98	0.89	1.05	0.54
		± 0.10 g	± 0.24 g	± 0.05 g	±0.08 g	± 0.04 g	± 0.21 g
	**IUGR?**	-	Yes	-	No	-	Yes
	**Fetuses with IUGR**	13%	73%	20%	37%	4%	61%
	**Mean fetal weight: Placental weight ratio**	9.0 +/− 1.0	4.7+/−2.3	9.5+/1.6	10.2+/−1.9	9.8+/−0.2	7.0+/−1.9
	**Mean size of the fetuses**	1.38	0.92	1.31	1.25	1.8	1.08
		±0.29 cm2	± 0.42 cm2	±0.20 cm2	±0.25 cm2	±0.14 cm2	±0.35 cm2
	**Mean weight of placentas**	0.10	0.08	0.11	0.08	0.11	0.07
		± 0.02 g	± 0.02 g	± 0.02 g	± 0.03 g	± 0.01 g	± 0.01 g

Pregnancy outcomes such as maternal weight and abortion showed that worst parameters were associated to P1 whereas P2 presented the best maternal parameters. P3 had an intermediate situation. Fetuses were counted on day of surgery (D0) and on termination day (E18.5) in control and test animals; mean litter size are presented. Fetal resorption rate is expressed as a percentage of fetuses counted at the time of surgery. Uterine artery ligation caused a significantly increased rate of fetal resorption in test animals from P1 and P3. P1 corresponded to the worst fetal viability rate and P2 to the best rate. IUGR was effective in P1 and P3 where more than 50% of fetuses completed these conditions. Fetal weight : Placental weight ratios were significantly reduced in test animals from P1 and P3. Measured fetal sizes were in accordance to recorded fetal weights with significant differences in P1 and P3. Placental weights were significantly reduced in all protocols. * D0 = E16 for P1, E17 for P2 and E16.5 for P3.

### Fetal characteristics

#### Number of fetuses and fetal resorption

The number of fetuses was determined both at day of surgery and day of extraction (E18.5) (Table 1); results reflected the rate of abortion and resorption observed in each protocol. In P1, the mean number of fetuses per mouse (litter size) was identical in control animals (8) and reduced in test animals (7 to 4). Similar results were obtained in P2 (7 mean number of fetuses in control animals *vs*. 7 to 6 fetuses found in test dams). In P3, mean number of fetuses found at E18.5 was significantly lower in test mice with 9 fetuses at D0 *vs.* 4 at termination. Control conditions led to similar number of fetuses at surgery and extraction (8). Thus, litter size was reduced in test mice. Fetal resorption was also precisely analyzed by comparing the number of fetuses found at the day of surgery and the number of fetus alive at E18.5. P3 presented the highest rate of fetal resorption with 37%, while P2 resorption rate was 0% (Table 1). P1 revealed an intermediate situation with 25% of global fetal resorption. Control groups didn’t show any fetal resorption.

#### Fetal viability

**Figure 3:**
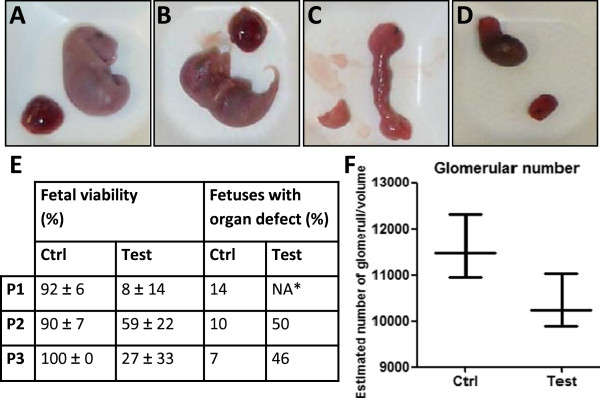
**Viability of fetuses found at extraction. A-D** Macroscopic observation of fetuses. Examples of viable/non viable fetuses and associated placentas are presented. **A**. Probably viable control fetus with pink/purple coloration and no visible malformation; **B**. Probably viable test fetus with pink/purple coloration and no visible malformation; **C**. Not viable test fetus with soft consistency and body malformation; **D**. Not viable resorbed test fetus. **E**. Mean fetal viability rates and macroscopic observation of organs. Mean fetal viability was assessed according to macroscopic observation of fetuses and are presented as mean value of data obtained from the different mothers used in each protocol; test animals were associated with lower viability rates. Macroscopic observation of the organs (kidney, liver, spleen, heart, lungs) was based on the following parameters : shape, coloration, consistency, differentiation and presence of blood spots. Macroscopic organ defects were analyzed and recorded from all control and test mice from each protocol ; * results from test mice from P1 are not presented as not relevant (only 7 fetuses were studied). Around 50% of test fetuses from P2 and P3 presented organ defects. *NA, Not applicable*. **F**. Mean number of glomeruli from control and viable test kidneys in P3. Estimated number of glomeruli in a total volume of 49,6 μm^3^ from P3 viable fetuses showed a tendency to decrease in test kidneys compared to control samples.

Fetal viability at termination day was also evaluated using macroscopic observations such as coloration, body consistency (normal, dry or soft), or body malformation (Figure 3A-E). Majority of not viable animals corresponded to resorbed fetuses (or in way of resorption) which generally showed abnormal coloration (dark, green *vs*. pink/purple), soft consistency and general body malformation (Figure 3D); other not viable fetuses were not resorbed but displayed abnormal consistency and body malformation such as head malformation, body length or absence of members (Figure 3C). As expected, control groups were associated with high viability rate (89.7 to 100%) and didn't show macroscopic abnormalities of the fetuses while test groups presented lower viability (Figure 3E). In P1 experiment, only 8% of the test fetuses were viable, compared with 59% for P2 and 27% for P3.

#### Observation of fetal organs

Macroscopic observation of fetal organs (kidney, heart, lung, liver) gave results in accordance with viability rates. Organs from control fetuses from all protocols globally didn't show macroscopic abnormalities, although observation of organs from test animals showed defects such as friable consistency, unusual coloration, blood spots, malformation or visible lack of differentiation. Data recorded for P2 and P3 (Figure 3E) showed that 46% (P3) to 50% (P2) of test fetuses found at termination presented such defects at different levels of severity compared to 7% (P3) to 910% (P2) of control fetuses. Most of these defects were visible on organs from non viable fetuses. In order to explore the impact of ligation on organs from viable fetuses, we observed kidneys from P3 control and test fetuses that also presented higher weights than the average. Glomeruli were counted and corresponding data showed a tendency (*P* = 0.2) to a 10% decreased number of glomeruli in kidneys from viable test fetuses compared to control fetuses (Figure 3F).

#### Weight and size of fetuses

**Figure 4:**
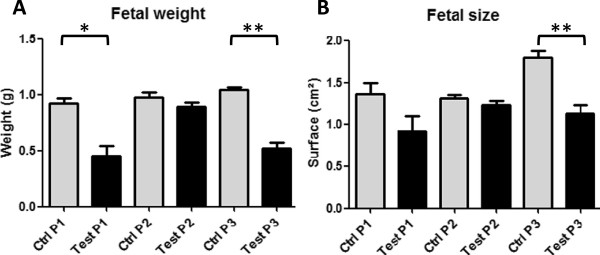
**Weight and size of the fetuses found at termination. A**. Weight of control and test fetuses from all protocols were recorded, significantly reduced weight was observed in test animals from P1 and P3. Mean weight of test fetuses from P1 and P3 completed IUGR definition (2 SD below the mean weight of control fetuses). **B**. Size of control and test fetuses from all protocols were measured and significantly decreased surfaces for test animals in P1 and P3 were recorded.

Weight and size of fetuses were measured in all experimental groups and presented significant differences between control and test animals in protocols P1 and P3 (Figure 4A-C). In P1, mean weight of test fetuses was significantly lower than the values of control animals (0.45 *vs.* 0.92 g, *P* < 0.05). Similar results were obtained with P3 (0.54 *vs.* 1.05 g), this difference was more significant in P3 than in P1 (*P* < 0.005). Nevertheless, the mean weights of fetuses in P2 didn’t reveal significant differences between control and test groups (*P* = 0.21). Thus, mean weight of test fetuses in P1 and P3 corresponded to IUGR conditions (2 SD below the mean weight of control fetuses), contrary to results of P2. In addition, 73% and 61% fetuses were growth restricted in P1 and P3, respectively, while only 37% were growth restricted in P2 (Table 1).

Sizes of the fetuses were in accordance with the measured weights: no significant reduction was observed in P2, while decreased surfaces were observed for test animals in P1 and P3, which also presented statistical relevancy (*P* < 0.005 – Table 1 and Figure 4B).

#### Position of ligation and influence on weight

**Figure 5:**
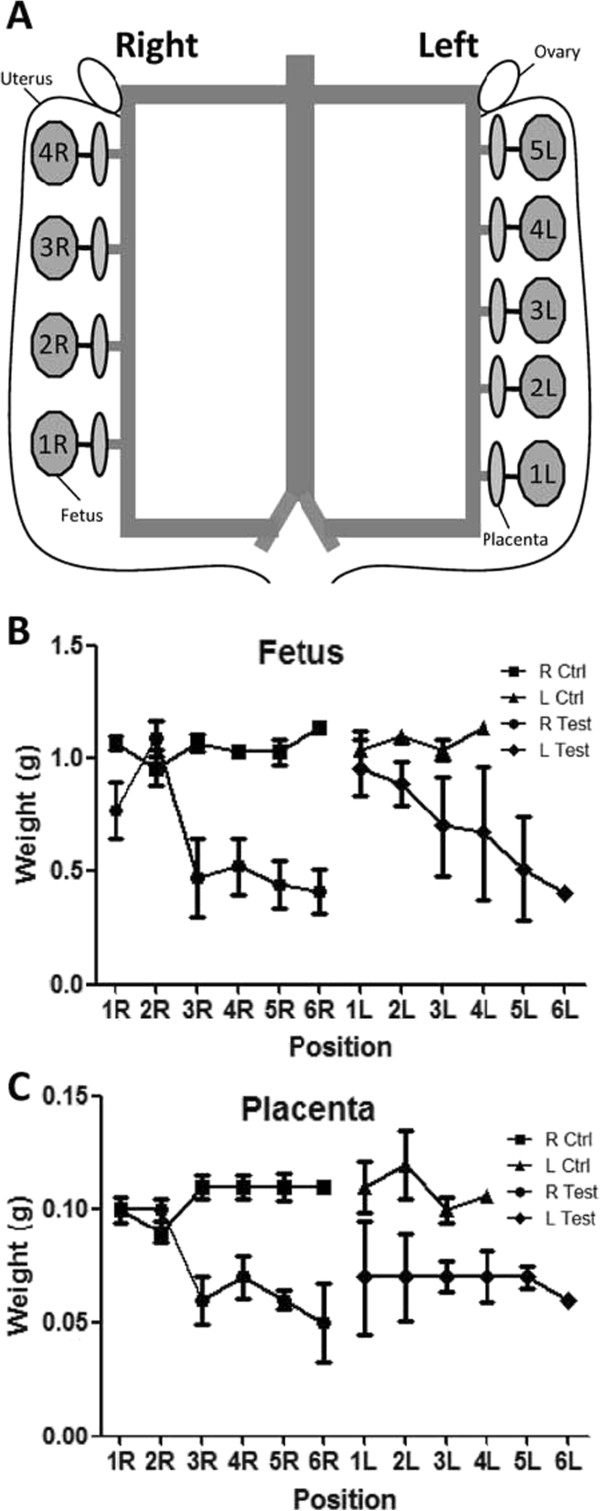
**Influence of ligation position on fetal weight. A**. Fetus numbering according to position in the uterus. Fetuses are numbered starting from the most caudal position in each uterine horn. R, right uterine horn; L, left uterine horn. **B**. Mean fetal weight according to position in the uterus in P3. More distal fetuses presented lower weights than caudal fetuses. **C**. Mean placental weight according to position in the uterus in P3. Placentas associated to distal fetuses presented lower weights in the right uterine horn, data from left side didn’t show significant differences.

Weight of fetuses from P3 (which significantly led to IUGR with good maternal and fetal viability characteristics) was also observed according to their position in the uterus in order to see the influence of ligation (Figure 5). Despite the different number of fetuses from the different test animals, fetal weight was decreased for fetuses placed after ligation (generally between fetus n°2 and 3) (Figure 5B). The same result was observed for placentas of the right side of the uterus from test mice, although left test placentas all showed a decreased weight (Figure 5C).

#### Weight and macroscopic observations of placentas

**Figure 6:**
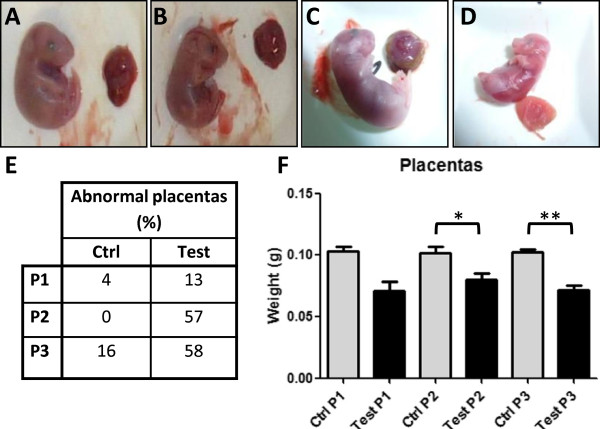
**Macroscopic observations and weight of placentas. A-D** Macroscopic observation of placentas: examples of placentas and associated fetuses. **A**. Normal control placenta associated to a probably viable fetus with central red coloration and peripheral beige coloration; vascularization is easily visible. **B**. Normal test placenta associated to a probably viable fetus with central red coloration and peripheral beige coloration; vascularization is easily visible. **C**. Abnormal coloration of a test placenta with brown peripheral color, associated to a probably viable fetus. **D**. Abnormal clear-pink colored placenta from a no viable test fetus. **E**. Placental abnormalities rates. Macroscopic placental defects were calculated from all placentas from P1, P2, P3; test fetuses were associated to a higher rate of abnormal placentas than control ones in all protocols. **F**. Mean weight of placentas from control and test animals in P1, P2, P3. Test placental weights were significantly reduced in all protocols.

As IUGR is associated with placental insufficiency, we also measured the weight of placentas at E18.5 (Figure 6). Interestingly, all protocols led to differences in placental weights, with P2 and P3 being associated with statistical significance (P2, *P* < 0.05, P3, *P* < 0.005, Figure 6). This was in accordance with reduced fetal weight and size in P1 and P3 protocols. Interestingly, P2 was also associated with a significantly reduced placental weight, although mean fetal weight didn’t complete IUGR conditions. Mean Fetal weight : Placental weight ratios (F/P) were calculated as a measure of placental efficiency and IUGR. F/P ratios were significantly reduced in test groups in P1 and P3 (*P* < 0.05, Table 1), with the lowest values associated to test animals in P1 (F/P =4.7). No significant differences were observed in P2. Macroscopic observations also showed differences between test and control groups, ligation was indeed associated to a higher proportion of placentas displaying macroscopic abnormal features such as unusual coloration (white, brown, black *vs*. dark red/pink) or lack of visible vascularization (Figure 6). Macroscopic abnormalities were mostly observed in placentas associated to non viable fetuses. In P1, 13% of the test retrieved placentas displayed macroscopic abnormalities *vs.* 4% in the control animals. In test animals from P2 and P3, 57% and 58% of the placentas were macroscopically abnormal, while only 0% to 16% of control placentas were affected (Figure 6).

## Discussion

Uterine artery ligation has long been used in large animals’ models such as sheep, rabbits or larger rodents to produce IUGR [8], nevertheless recent studies underline the weaknesses of this method. Notably, uterine artery ligation results in high abortion rate of fetuses (at least 30%, [8,10,11] and IUGR is not necessarily demonstrated [10]. Another problem of this commonly used surgical technique is that in rodents it is mostly performed on rats [8] and no studies have so far established it in mice. This animal model is however widely used by the scientific community, resulting in a huge availability of genetic models that are invaluable tools to study biological mechanisms and diseases. In the present study we developed a model of bilateral uterine vessels ligation in mice in order to study the biological mechanisms associated to IUGR. We set up three different protocols in order to obtain a model resulting in IUGR conditions with similar or improved health and viability parameters than what is usually observed in rodent bilateral uterine artery ligation studies. We then designed three different protocols with differences based on the position of the ligation and on the day of surgery. In Protocol 1 (P1), early surgery was performed at E16 with ligation around the cervical end of each uterine horn, while in Protocol 2 (P2) and 3 (P3), ligation was performed on E17 or 16.5 around the central part of each uterine horn. Performing a distal ligation of the uterine vessels near the cervical end of each uterine horn leads to a complete blocking of the blood flow from the iliac artery. The remaining blood flow comes only from the ovarian arteries and is significantly reduced. On the contrary, when performing a ligation of the uterine vessels at the central part of each uterine horn, the blood flow from both the iliac and ovarian arteries is still existing. The restriction of blood flow is then lower than the one induced by a distal ligation. The results we obtained concerning both the follow-up of pregnant operated mice and the weight of their fetuses underlined the importance of surgery location and time-point parameters. As expected, an earlier and more caudal surgery had a higher impact on fetal weight, but this was also accompanied by higher abortion rate and high mortality of the mothers, with bad viability prognostic for the fetuses (Table 1). In P1, which led to IUGR of the fetuses, we indeed observed a strong impact of surgery on the health status of test mice which led to death or euthanasia of 50% of them. We then improved our protocol by changing ligation site and time of surgery. The resulting protocols P2 and P3 gave much better results in terms of maternal parameters, but only P3 was associated with IUGR of the fetuses. Then, in P3, no prenatal maternal mortality was described while fetal weight was significantly decreased.

Time of surgery itself has an important impact on the health status of the operated mice, as indicated by the weight differences observed between control animals. Surgery had an important impact on mice health status, as weight loss was observed for all the groups (test and control from all protocols). Thus, in P2, a shorter recovery time (1.5 days) led to a decreased weight for both test and control pregnant mice, while control mice from P1 had more time to recover (2.5 days) which resulted in a weight gain one day after surgery. In the same way, control mice from P3 protocol started to gain weight at the end of the experiment (two days after surgery). For all protocols, control mice underwent a weight loss just after surgery, thereafter they started to recover or stabilize depending on the length of recovery. In this case, early weight loss was slight and was not associated with abortion. For test animals, all protocols led to a decreased weight which was associated with ligation. Notably, P1 was associated with the biggest difference between control and test animals, and was also associated with the worst viability parameters. Such consequences are to be related to ligation itself, as control animals from P1, which underwent sham procedure, recovered. Weight loss of test animals was comparable in all 3 protocols (−18 to −22% of body mass, Table 1), and underlined the effect of uterine artery ligation during pregnancy. This decrease can notably be attributed to partial abortions and partial deliveries observed in test groups, while weight loss from control groups in P2 and P3 can be linked to short recovery times after surgery. High weight loss and mortality rate from P1 test animals led us to change our protocol in P2 and P3 to a shorter experimental time.

Another important experimental point is the rate of abortion encountered with uterine artery ligation, leading to reduced litter size. Here, we report that all test mice from P1 underwent partial abortion, while 75% test mice from P3 did (Table 1), underlying strong impact of surgery on mice from P1. In P2, no abortion was observed. In the same way, P1 was accompanied by the highest resorption rate, as opposed to P2. In this regard, P3 seems to be an appropriate intermediate leading to IUGR while resulting in similar resorption rates to what is observed with rat model [10,11,15] and higher viability than P1 (Table 1). Not surprinsingly, P2 presented the best viability rate. Nevertheless, both abortion and resorption rates in P3 still led to a significantly reduced number of fetuses found at termination, representing a loss of 50% of fetuses, a parameter that has to be taken into account to set up further ligation experiment. In particular, results showed variability in the resorption rate (Table 1). This was due to the differences observed between test animals, with for instance 2 animals having all fetuses resorbed while 4 didn't show any resorption. The differences in viability data also show the differential impact of ligation at E18.5 depending on the protocol; the resulting viable fetuses can be used to study the mechanisms and consequences of IUGR after birth. Further studies could also take into account the possible additional mortality that can occur during labor in case of IUGR.

During pregnancy, placenta supplies maternal substrates to the fetus and can adapt to the maternal environment with changes in substrate delivery to the fetus [16]; it thus plays an important role in fetal programming *in utero* and placental weight is related to birth weight and fetal growth [17]. Placenta is notably very vulnerable to a decreased blood supply. All protocols illustrated this fact with lower test placental weights following surgery and statistical significance for P2 and P3. Due to less test animals available in P1, results didn't reach our significance threshold. Despite this observation, IUGR was not always associated to a decreased placental weight, as P2 didn’t reach these conditions (mean weight of test animals 2 SD below the mean weight of control animals), which could be due to the shorter exposition time to decreased blood supply compared with the other protocols and in particular P3 (E17 to E18.5 *vs.* E16.5 to E18.5). Thus, in P3, placental weight was more significantly decreased than in P2, which paralleled a higher impact on fetal growth and underlines the influence of the placenta on birth weight [16]. In order to study placental efficiency, F/P ratios were calculated as they also constitute an important indication of IUGR. Our results showed decreased F/P ratios in test groups from P1 and P3, with no significant differences in P2. These results are in favor of placental insufficiency and IUGR in P1 and P3. Indeed, a decrease in F/P ratio indicates that IUGR is linked to a reduction of placental function per g of tissue and increasing severity of IUGR has been shown to be associated with increasing P/F ratio at comparable gestational age (and thus decreasing F/P ratio) [18,19]. In case of IUGR, placental size is affected before fetal size and more resources are invested in fetal growth than in placental growth. In our study, ligation occurs at late gestation, when mouse fetuses are normally growing most rapidly [20]. Thus, the impact of limitation is very important and IUGR occurs when placental mass per g of fetus becomes critical. In P1, in addition to a distal position, ligation was made earlier than in other protocols, at E16. F/P ratios from test group in P1 are also the lowest ones, while P2, which corresponded to the shortest experimental time (ligation at E17), didn't present significant differences. Only placental weights are decreased. Nevertheless, the slight -but not significant- increase in F/P ratio in P2 associated with the decreased placental weights could suggest a better efficiency of these placentas to maintain fetal growth. P3 had an intermediate situation with an experimental duration leading to IUGR and a decreased F/P ratio for test animals. As fetal growth is more important at the end of gestation, the severity of the impact of ligation increases with the duration of the experiment. Delaying surgery from E16.5 to E17 thus leads to IUGR and decreased efficiency of placentas in P3 and not in P2. In addition, in test animals from all protocols, ligation was associated to macroscopic abnormalities of the placentas. This parameter reflects the impact of surgery on placenta condition and efficiency and is related to viability of the fetuses.

Impact of ligation and corresponding decreased blood supply was also visible on test fetal organs (kidney, heart, lung, liver) that displayed macroscopic abnormalities. These defects, such as malformations or lack of differentiation, can be related to the consequences of IUGR and an adverse uterine environment in adulthood as a risk factor for several diseases such as hypertension, cardiovascular problems, insulin resistance or end stage kidney disease. As a consequence, additional data like weight of organs could provide interesting information in order to further characterize this IUGR model. In particular, this could give information on the putative differential growth of organs that can favor organs at the expense of others that occurs in different cases of IUGR [1]. In our study, macroscopic defects were mostly observed on non viable fetuses (Figure 3). We thus also analyzed the impact of ligation on the kidneys of viable fetuses by counting glomeruli from P3 animals using morphometric methods. We indeed observed a difference between the number of glomeruli in test kidneys compared to control organs. Due to the variability of this *in vivo* experiment, the number of available animals, the technique used and the fact that the analyzed fetuses had a higher weight than the average, this result didn't reach our significance threshold (*P* = 0.2 *vs. P* < 0.05), nevertheless it showed a clear tendency towards a decreased number of glomeruli in test kidneys (10%, Figure 3F) and thus a probable defect of kidney differentiation. As nephrogenesis ends at D7 in mice, this difference could be more important at the end of this process.

Our experiment showed that uterine artery ligation was effective to induce IUGR. Fetal weights were measured at termination day E18.5 in order to avoid heterogeneity resulting from different delivery time. Our data reveal significantly decreased weights for fetuses from test animals of P1 and P3 (Table 1). P1 presented the most growth restricted fetuses, with mean weights of 0.45 g vs. 0.66 g for P3, but was also associated with the worst pregnancy outcomes parameters. P2 led to tendencies of growth restriction but without statistical significance (Table 1). Not surprisingly, ligation position in P3 was shown to influence the weight of fetuses depending on their position in the uterus, which was also responsible for variation in the recorded weights, although still lower than control fetal data (Figure 5). Globally, placental and fetal weights were not significantly different between left and right horns. In P3, we ligated vessels in the central part of each uterine horn, most often after fetus number 2. In the right horn, placentas and fetuses situated after ligation presented decreased weights compared to first fetuses. In the left side, same tendency seemed to be observed for fetuses, however experimental variations in small weights made it hardly detectable for placentas. In normal conditions, placental and fetal weight variations can be observed depending on the number of fetuses in the horns and their position. The larger the litter, the smaller each fetus tends to be [17]. In particular, the quantity of nutrients available for fetuses depends on the pressure at which blood reaches the placenta. Less fetuses were present in the left horn (mean 4 *vs*. 5, data not shown), which could explain that placental and fetal weights are somewhat more important than on the right side, but the difference is not significant. Variations in placental and fetal weights due to litter size and position (due to blood gradient) are amplified by ligation. Sizes of the fetuses also reflected intrauterine growth restriction, with decreased surfaces for fetuses from operated animals in P1 and P3.

## Conclusions

This method of bilateral uterine ligation in mice is efficient to induce IUGR. As a consequence, our study shows for the first time the feasibility to use mice as a model of IUGR/placental deficiency with the technique of bilateral uterine ligation. Surgery according to the protocol P3 enables to study IUGR with good maternal parameters and similar resorption and viability parameters to what is observed in rat models. Thus, the novel surgical protocol we established here paves the way to study molecular mechanisms of IUGR and test various treatment options, with the benefit of a huge availability of biological tools dedicated to murine models.

## Competing interests

The authors declare that they have no competing interests.

## Authors’ contributions

UHD conceived the research. MJ, MLCD and UHD designed the project. MLCD conducted the animal experiments and acquired the data. MJ and SR conducted tissue experiments and SR acquired glomerular data. MJ and UHD interpreted the data. MJ drafted the manuscript. MJ, MLCD, SR and UHD revised it critically for important intellectual content. All authors read and approved the final manuscript.
